# Design of a multi-category drug information integration platform for intelligent pharmacy management: A needs analysis study

**DOI:** 10.1097/MD.0000000000037591

**Published:** 2024-04-12

**Authors:** Qihong Pan, Yang Liu, Shaofeng Wei

**Affiliations:** a College of Traditional Chinese Medicine, Nanchang Medical College, Nanchang, China; b Organization Department of the Party Committee, Nanchang Medical College, Nanchang, China; c College of Pharmacy, Nanchang Medical College, Nanchang, China; d Key laboratory, Jiangxi University of Chinese Medicine, Nanchang, China.

**Keywords:** drug, information, integration, management, pharmacy

## Abstract

A drug store was never just an area to fill personal solution. Patients considered drug specialists to be counsels, somebody who could help them pick an over-the-counter treatment or understanding the portion and directions for a solution. Drug stores, similar to the remainder of the medical services business, are going through changes. Nowadays, one of the main highlights of any structure is the board. The executives give the refinement needed to wrap up any responsibility in a particular way. The executive framework of a drug store can be utilized to deal with most drug store related errands. This report has provided data on the best way to fabricate and execute a Pharmacy Management System. The primary objective of this system is to expand exactness, just as security and proficiency, in the drug shop. This undertaking is focused on the drug store area, determined to offer engaging and reasonable programming answers to assist them with modernizing to rival shops (helping out other equal modules in a similar examination program). This study will clarify the system’s thoughts concerning the board issues and arrangements of a drug store. Likewise, this study covers the main parts of the Pharmacy application’s investigation, execution, and look.

## 1. Introduction

The board framework of a drug store is a kind of executive framework that is expected to further develop the exactness of the drug store, just as the wellbeing and proficiency. As a framework based on personal computer (PC), the board framework of a drug store helps drug specialists in further developing stock administration and cost, clinical security, and other things. During an initial stock and deal exchange, the framework permits the client to enter an assembling and expiry date for a particular item or medication. Likewise, the framework will create a report that shows a rundown of items that are going to terminate after a specific date. Additionally, the framework requires manual section upon the appearance of new groups of medications and upon drug development out of the drug store for a set timeframe, like consistently. The drug specialist might need to create a report for the development of medications all through the drug store, acquiring data about the medications, for example, expiry date, date bought, number of medication types left, and the area of a medication in the drug store.

Any framework utilized in a drug store to assist the mechanization of the drug store work is known as executive programming. This incorporates undertakings like inspecting doctor orders and planning prescriptions, monitoring stock and putting in drug requests, charging and protection, advising, distinguishing inconsistencies, and that’s just the beginning - all while sticking to lawful conventions and guidelines.

These are only a portion of the normal assignments that can be robotized. A lot more highlights can give the drug store an upper hand by further developing client support and drawing in patients with more customized and connecting with administration. In the accompanying area, we’ll go over these highlights in more prominent profundity. Presently, attention should be paid to the key benefits that the executive framework of a drug store might give.

The effectiveness of drug specialists will be improved. Drug specialists invest most of their energy in apportioning medications. The medication assignment requires extreme fixation, broad confirmation, drug cooperation checks, and interpreting the specialist’s penmanship. Is it important to physically apportion drugs? Not at all. Solutions might be promptly overseen by programming through setting up impeccable correspondence from PC to PC, giving drug specialists more opportunities to meet with patients. This carries us to the following benefit. Drug specialists are allowed to work on the soundness of patients. Patients look for exhortation from drug specialists, and a pharmacy management system can provide some help in terms of improving counsel, either straightforwardly or by implication. Drug specialists can talk with clients online through a patient entry, as well as investing more energy with them face to face. Moreover, drug specialists can get to a patient’s prescription history to improve suggestions by referring to a medical clinic’s electronic health record. Besides, unique medication adherence devices can help patients deal with their drugs by permitting them to effortlessly reorder refillable medicines and get warnings about these medicines.

The extortion in the medicine business will be forestalled. All solution data is imported into the Prescription Drug Monitoring Program information base so that the drug administration can be checked. Notably, drug stores assume a basic part in assisting with dealing with the dispersion of controlled hazardous substances. As data is consequently added to the experience set of patients, the board framework of drug stores incorporated with the Prescription Drug Monitoring Program gateway permits drug specialists to diminish logging time and work to only a couple of clicks.

## 2. Features of a pharmacy management system

Drug specialists utilize a few distinct sorts of PC frameworks.

• Requesting frameworks that are available on the web. These frameworks, most of which are provided by drug wholesalers, permit drug specialists to arrange prescriptions straightforwardly from the distributer’s site.
• Frameworks that monitor stock endlessly. According to the government law, ceaseless frameworks (computerized or not) are used for Schedule II controlled substances, which involves consistently recording the amount of medicines as the remedy is filled and apportioned. Accordingly, the drug is consequently taken out of stock, and the stock data is cutting-edge 100% of the time.
• Frameworks that administer corresponding work. These frameworks are machines that count and administer pills for a drug specialist. A few more modern frameworks will even print the mark and apply it to the jug.
• A pharmacy management system, for the most part, replaces an interminable stock framework and adds capacities and connectors to deal with any remaining exercises.

## 3. Review of related studies

### 3.1. Intelligent pharmacy management

The application of man-made consciousness in drug innovation has increased lately, and it might not only set aside time and cash but also additionally further develop information on the connections between different plans and cycle factors. Man-made reasoning is an area of software engineering worried about critical thinking by means of representative programming. Actually, man-made reasoning has developed into a critical thinking science with various applications in business, medical services, and design. In this paper, we examined drug disclosure, artificial intelligence (AI) apparatuses, manufacturing execution system frameworks, automated communications processing system, AI to anticipate new medicines, advancement of novel peptides from regular food varieties, treatment and the board of uncommon illnesses, drug adherence and measurement, and boundaries to AI reception in the drug business.^[1]^

The objective of this study is to find a proper and forward-thinking board framework of clinic drug store for Bangladesh.^[2]^ The drug store area is viewed as the core of each clinic since it is connected with offices like medical procedure, cardiology, nephrology, medication, pediatrics and others. In spite of the fact that Bangladesh’s drug business has developed fundamentally, and its items are of good quality, the erroneous administration framework in medical clinic drug stores still brings the affliction to patients. Therefore, emergency clinic drug store improvement is important to guarantee the accurate identification, arrangement, store, compounding, and administer of medication and clinical hardware, just as advising for patient security and consistence (Bangladeshi Hospital Pharmacy Management System and Future Development Approaches).

AI is next outskirts of pharmacy in bio sciences. In this study, we inspected the most current AI systems pointed toward mimicking scholarly capacities of humans, such as AI and Robots. “Computerization turned into the outcome of Industrialization,” persuaded by the longing to help creation, accomplish uniform quality, and free workers from hazardous and weighty errands.^[3]^ PAT, CFD, and Pharmaceutical Automation in R&D are ongoing AI progresses in drug stores. These AT techniques provide broad data support with respect to approaches that have recently been utilized in medical care, such as inhaler plans, drug ingestion and disintegration, and illness situations. Overall, the robot for drug applications has a splendid prospect. With a maturing populace that earnestly requests refined clinical contraptions and fresher drugs, mechanical technology frameworks are progressively being utilized to fulfill people’s needs for improving medical usefulness and proficiency. Producers of robots, then again, go up against various impediments in their endeavors to lay down a good foundation for themselves in drug applications. The utilization of AI and robots in human existence has various advantages and downsides. Regardless of the developing collection of AI writing, AI strategies, for example, artificial neural network, AI, AI in medical services, and AI in clinical practice keep on being utilized. This study centers around a couple of sickness difficult to solve in reality: malignant growth, sensory system infirmities, and cardiovascular infections. What’s to come is hard to anticipate, yet AI will shape it, as it will be the following boondocks in a drug store.

AI and man-made consciousness, the eventual convergence of each discipline, can play an improving or promoting role in any area. Both AI and man-made consciousness might be utilized in a retail drug store to address an assortment of issues. The AI expectation model can be applied to identify the patients’ infection and anticipate their prescription. AI frameworks might be utilized to mechanize processes, accumulate time-reserve funds and reduce the consumption of assets.^[4]^

Drug Store Leadership and Management is a 12-day concise experiential learning recreation for conclusive year understudies. Understudies in six-man groups deal with an essential consideration drug store and are given about 180 situations and north of 400 medication related errands.^[5]^ This program depends on the GIMMICS consortia of foundations’ effective methodology. To evaluate the worthiness and utility of the program for understudies and their future work, 221 understudies in the module got an internet-based 82-thing survey with open and shut inquiries on their module encounters. There were 2 updates given. Recurrence rates were utilized in the examination. Specifically, 65% of understudies (n = 143) reacted to the survey. Among them, 92% of understudies (n = 132/143) expressed that the module tested them, 89% thought it expanded their certainty while talking with patients (n = 127/143), 84% (n = 110/131) felt their group working capacities improved as the module proceeded, and 89% (n = 117/131) said the module assisted them with solidifying their data all through their certification. Notwithstanding the idea of the program, the greater part of the understudies (55%, n = 71/131) accepted that the business abilities they mastered would be futile in their future vocations. Collectively, this new module has supported understudies in their self-improvement by reinforcing their cooperation capacities. In spite of the need to learn business and the board, understudies quite often need information regarding the matter, perhaps because of an absence of functional openness in the course. This is the main year the module has been offered, and we will be circling back to the now-ongoing alumni to perceive what the educational plan has meant for their profession movement.

## 4. Multi-category drug information integration platform

Drug-Target Interactions (DTIs) constitute a significant piece of the medication disclosure process. It is expensive and tedious to recognize DTIs with conventional exploratory techniques. Fortunately, computational methodologies offer an assortment of successful techniques for managing this issue. Most of computational strategies for recognizing DTIs have depended on data, for example, drug-drug comparability or target-target similitude as of late, which doesn’t impeccably catch all qualities.^[6]^ We proposed another computational model for DTI forecast in light of AI strategies in this paper. On the one hand, we utilized atomic base fingerprints, Multivariate Mutual Information of proteins, and organization geography to address medications, targets, and their connections to further develop expectation execution. On the other hand, we utilized the Support Vector Machine and Feature Selection calculations to assemble a model for foreseeing DTIs. Tests uncovered that the proposed procedure beat existing driving methodologies for foreseeing highlight based DTIs. On the Enzyme, Ion Channel (IC), GPCR, and Nuclear Receptor datasets, the proposed technique accomplished areas under the precision-recall curve (AUPRs) of 0.899, 0.929, 0.821, and 0.655, individually. On Ion Channel datasets, AUPRs were expanded by 0.016 when contrasted with existing best techniques. Besides, our strategy accomplished the subsequent best outcomes on GPCR and Enzyme datasets.

Unfriendly drug-drug interactions (DDIs) are significant in drug advancement since they are the main source of dreariness and passing. In fact, recognizing DDIs is basic for specialists, patients, and society. Traditional AI strategies rely upon hand-tailored attributes essentially and need over-simplification. Profound gaining techniques that can gain drug attributes from an atomic chart or medication related organization have as of late improved PC models’ ability to foresee obscure DDIs. Previous exploration, then again, depended on enormous named datasets and zeroed in exclusively on drug design or grouping data, disregarding the connections or topological data among drugs and other biomedical articles (e.g., quality, illness, and pathway), or on information diagram (KG) without considering data from the medication atomic construction. Therefore, MUFFIN, a multi-scale feature fusion deep learning model, was proposed in this study to effectively analyze the joined impact of medication atomic design and semantic data of drugs in information diagram on DDI prediction. Biscuit can become familiar with a medication portrayal in view of both medication self underlying data and KG with broad bio-clinical information. To combine multi-modular highlights effectively, we made a bi-level cross methodology in MUFFIN that joins cross-and scalar-level parts. By crossing over the qualities gained from huge scope KG and medication sub-atomic chart, MUFFIN might facilitate the impediment of restricted marked information on profound learning models. We tried our technique on 3 datasets and 3 particular DDI forecast errands: 2-fold class, multi-class, and multi-mark. Biscuit outflanked other cutting edge baselines, as indicated by the discoveries.^[7]^

Most organizations prefer to build programming arrangements like cloud stages using an open-source methodology.^[8]^ In any case, mistakenly incorporating assorted programming parts may bring about stages that need suitable non-useful characteristics and fail to develop to deal with an extension in customer base. It’s worth noting that such frameworks would not be able to contend successfully in the cloud. A progressive procedure to decide the best attainable incorporation technique and methodology for cloud stages was proposed in this study, considering the goal of prior issues and the formation of stages that can beat the opposition. This procedure depends on analyzing the current open-source stage parts to be combined and choosing the ideal incorporation technique and system that yields the best conceivable joining result while requiring minimal measure of mixing work. How this procedure deals with the MELODIC multi-cloud the executives stage, which is currently well known in the cloud business, was described in this article.

Drug exercises on a multi-scale, from nuclear complexities of medication target connections to new elements of natural organizations, are the objective of frameworks pharmacology, which attempts to sanely foster medicines that focus on an associating network rather than a solitary quality.^[9]^ Complex information driven examinations, including prediction based on AI, are common in frameworks pharmacology. Mix of various omics information is the first stage in quite a while, followed by enhancement and prediction. The general methodology for drug-target affiliation prediction utilizing REMAP, a huge scope off-target expectation device, was portrayed in this paper. The technique displayed here can be utilized to address different difficulties in frameworks pharmacology, including connection surmising.

It is reported that drug-drug interaction databases (DIDs) differ basically with respect to classification of DDIs. The objective of this review was to investigate the 5 most generally utilized open-access English language-based internet based DIDs and the 3 most ordinarily utilized membership English language-based web-based DIDs in writing for straightforwardness of possession, financing, data, characterizations, staff preparing, and hidden documentation. In the clinical writing, we directed a deliberate writing search to distinguish the 5 most regularly utilized open access and the 3 most normally utilized membership DIDs. Proprietorship, arrangement of cooperation, essential data sources, and staff capability were completely assessed for every one of the information bases. Fisher’s accurate test was applied to analyze the general extent of yes/no responses from open access and membership information bases when mentioning missing information. The analysis outcomes exhibited that 20/60 open access DIDs could be checked straightforwardly from the page, contrasted with 24/36 membership DIDs (*P* = .0028). These figures were expanded to 22/60 and 30/36, individually, after an individual solicitation (*P* < .0001). The extents were 3/25 versus 11/15 accessible from the site page (*P* = .0001) and 3/25 versus 15/15 (*P* < .0001) accessible upon individual solicitation for things in the “order of association” area. All in all, data accessible online with regards to proprietorship, financing, data, arrangements, staff preparing, and hidden documentation shifts essentially between DIDs. On the boundaries tried, open access DIDs had a measurably lower score.^[10]^

DrugBank is a unique bioinformatics/cheminformatics information base that unites itemized drug (for example synthetic) information with broad medication target (for example protein) information.^[11]^ There are about > 4100 medicine sections in the information base, including over > 800 FDA-supported little atom and biotech drugs and over > 3200 investigational drugs. What’s more, these prescription sections are associated with about > 14 000 protein or medication target successions. Each DrugCard section has about > 80 information fields, with half of them committed to tranquilize/compound data and the other half to medicate target or protein data. Numerous information fields interface with outer data sets (KEGG, PubChem, ChEBI, PDB, Swiss-Prot, and GenBank) and construction seeing applets. The information base might be looked at in an assortment of ways, including text, grouping, substance structure, and social inquiries. The major potential uses of DrugBank consist of silico drug target recognition and proof, drug configuration, drug docking or screening, drug digestion forecast, drug communication expectation, and general drug schooling.

Recently, quality articulation has been demonstrated to be the most helpful information for foreseeing drug reaction. Ongoing proof recommends that fusing more omics can further develop expectation precision, which makes one wonder how to do as such. Clinical utility and translatability are basic, no matter what the joining procedure.^[12]^ Subsequently, we theorized that joining multi-omics with clinical datasets would further develop drug reaction prediction and clinical pertinence. MOLI is a multi-omics late integration strategy in view of profound neural organizations we proposed. MOLI takes information from physical change, duplicate number deviation, and quality articulation and joins it to anticipate drug reaction. Besides, MOLI learns highlights for each omics type utilizing type-explicit encoding sub-organizations, connects them into one portrayal, and streamlines it utilizing a joined expense work comprising of a trio misfortune and a paired cross-entropy misfortune. The previous makes responder tests’ portrayals more like each other and recognizes them from non-responders, while the last option makes this portrayal prescient of reaction esteems. As for 5 chemotherapy specialists and 2 designated therapeutics, we approved MOLI on in vitro and in vivo datasets. MOLI presents higher prediction exactness in outer approvals than cutting edge single-omics and early incorporation multi-omics strategies. Moreover, preparing on a skillet drug input, for example, utilizing all drugs with a similar objective, further develops MOLI’s presentation for designated medicates fundamentally more than preparing on drug-explicit data sources. The high prescient force of MOLI indicates that it can very well be helpful in accuracy oncology.

In the post-genomic period, organic high-throughput advances like proteomics and transcriptomics have produced an expanding measure of information. This omics information helps a lot, yet the genuine test is to dissect all of the data that has been incorporated. Questioning unique, heterogeneous, and disseminated biomedical information sources is conceivable with biomedical information mix. With regards to medicate creation, in addition to biomedical data recovery, clinical diagnostics, framework science, and different regions, information integration

## 5. Design of a multi-category drug information integration platform

For better activity and accomplishment of hierarchical objectives, the associations should coordinate their divergent authoritative units, data innovation, and business processes. Managing assorted applications that use unmistakable structures (grammar) and join various implications (semantics) to information presents a test. There is a test in synchronizing work process with the end goal that the different authoritative units work as a brought together entirety. The tremendous idea of the venture joining challenge forestalls ways that handle the whole issue, and, on second thought, requires choices that emphasis on a particular yet important type of combination. However, a great many types of data mix and how they interface with each other remain to be explained. In this article, we proposed an endeavor data coordination system that attempted to unite unique strategies into combination to acquire a superior comprehension of the test. The endeavor data coordination structure distinguishes 4 layers of the venture framework, each with its own arrangement of boundaries and data mix sorts. The system is utilized to look at the current condition of corporate data reconciliation innovations just as possible arrangements.^[13]^ The review features expansive patterns just as examination holes that should be filled.

In multidisciplinary improvement, engineers utilize an assortment of space explicit instruments to indicate and examine a framework. Generally, productive framework advancement requires that the models created by these devices be very much coordinated into the general framework to decrease the danger of irregularities and clashes in the plan data determined. In 2005, El-khoury et al, revealed a design for the incorporation of model and apparatus. They assembled the essential parts of their design with the help of notable and perceived norms from both PC and mechanical design. Model coordination is upheld by engineering, which permits models working on independent devices for various parts of a similar framework to be connected so that the information can be shared and exchanged. Similarly, the coordination stage takes fine-grained model administration into account. Therefore, it can be inferred that a blend of highlights is presented in customary information of the board frameworks, such as item information executives (PDM) and programming design the executives (SCM).

Despite great advances in genomic, proteomic, and high-throughput screening approaches and the upgradation in sane medication plans, the quantity of creative, single-target treatments has missed the mark concerning assumptions during the last 10 years.^[14]^ By adding a scope of backhanded, network-subordinate impacts, there is an increase in multi-target medicines in their quantity of important pharmacological target atoms. Additionally, the low-proclivity restricting of multi-target prescriptions lessens druggability limits and drastically extends the druggable proteome. These impacts immeasurably increase the quantity of conceivable pharmacological targets and give new sorts of multi-target prescriptions with lower poisonousness and antagonistic impacts. The creators inspect different organization assault techniques and propose various ways of finding objective sets of multi-target drugs.

This work proposes a data coordination displaying design for item everyday life cycle (I(2)MAfPFL) to work with incorporating, making due, trading, and reusing data over the entire life pattern of an item family in mass customization. The I(2)MAfPFL is focused on the model of three-layered (three dimensional) state space with joining of the model of unit data and the model of the three dimensional development space as an engineering planned according to the point of view of the whole item family rather than a solitary item. The model of unit data is used in the I(2)MAfPFL to address the attributes of unit data made at various profundities and levels during all periods and daily life cycle of the item. The three dimensional state space model offers the design and structure for incorporating unit information. The three dimensional development space model portrays the unique advancement cycles of unit information and presentations the natural associated, connected, and developed connections among them. In the I(2)MAfPFL, the declaration of the three models is given top to bottom.^[15]^ Finally, a situation is given to show how the I(2)MAfPFL is used in mass customization to fuse item day to day life cycle data.

The Vienna Cloud Environment is a coordinated system to satisfy the need for connecting biomedical information through developing a general, administration based information foundation. The foundation is comprised of many help types that, in one situation, are utilized to uncover and connect various open medication and clinical preliminary information sources through a typical point of interaction.^[16]^ As a result, local area of the biomedical examination currently has a straightforward method for questioning medication related qualities, for example, nonexclusive compound names, brand names, or atomic targets. We make and investigate numerous methods for setting up such a joining foundation, just as the consequences of this structure utilizing a trial evaluation of a medication preliminary coordination application. The establishment and trial evaluation of the medication preliminaries mix application ignited some useful suggestions and hypothetical worries.

In recent years, progresses in computer-aided drug design techniques have sped the beginning phase of drug research.^[17]^ In scholarly community, numerous free computer-aided drug design innovations have been created. Since various examination associations are creating programs, a uniform and easy to understand online graphical work space is required, joining computational methodologies, for example, pharmacophore planning, likeness estimation, scoring, and target recognizable proof. Referring to pharmacophore and 3D atomic comparability, we gave an adaptable, easy to understand, and quick web-based stage for computer-aided drug disclosure. Through a smooth connection point to every changed bundle, the internet based point of interaction accomplishes restricting locales discovery, virtual screening and hitting of recognizable proof, and medication target prediction in an intelligent way (e.g., Cavity, PocketV.2, PharmMapper, SHAFTS). Additionally, iDrug incorporates numerous economically open compound information bases for hit distinguishing proof and particularly clarified pharmacophore data set for drug target expectation. Ongoing sub-atomic structure/altering, changing over, introducing, and examining are altogether conceivable utilizing the web-based point of interaction. Each of the utilitarian modules’ adaptable designs might be gotten by means of the offered, included meeting records, which can be saved to the neighborhood circle and transferred to continue or alter past work. iDrug is easy to utilize and gives a creative, speedy, and dependable device for drug configuration studies. iDrug allows clients to submit and look through different sub-atomic plan handling occupations in a solitary program without introducing any different demonstrating programming locally.

Drug Target Commons is a local area driven bioactivity information joining and normalized internet based stage (data set with UI) for exhaustive planning, reuse, and investigation of compound-target association profiles. End clients can use an application programmable point of interaction, data set dump, or tab-delimited text download choices to look, transfer, change, explain, and trade master organized bioactivity information for additional examination. Drug Target Commons 2.0 offers refreshed clinical improvement data for the medications and target quality infection associations, just as disease type signs for freak protein targets, which are urgent for accuracy oncology progresses.^[18]^

Biomedical exploration networks should share and coordinate information among their individuals just as with outside partners. A viable data innovation framework is needed to work with such information trade tasks. We made a reference model for the requirements to make the formation of such a framework simpler. There are 5 reference objectives and 15 reference prerequisites in the reference model. The objectives and prerequisites are determined in association with one another utilizing the Unified Modeling Language. Furthermore, all destinations and requirements are literarily communicated in tables. This reference model might be used by research networks as a reason for securing project-explicit prerequisites in an asset effective way.^[19]^ Moreover, an exploration network on liver disease is given as a particular illustration of the reference model. The reference model is changed into an organization’s explicit necessities model. A help situated data innovation engineering is made and clarified in this work in light of this sensible necessity model.

The advancement in medication development is high-cost and prone to disappointing outcomes. Reusing a medication made for one sickness to treat a subsequent infection is possibly less hazardous and costly than fostering an out and out new compound. To support throughput and find competitors all the more reliably, drug repositioning is required with the aid of orderly strategies. We addressed this prerequisite by fostering an incorporated frameworks science dataset for the in silico ID of novel medication repositioning up-and-comers utilizing the Ondex information combination stage.^[20]^ We showed how the data in this dataset might be utilized to observe realized repositioning cases. Moreover, a robotized strategy was proposed to track down original treatment signs of current medications in this paper.

Drug methods have advanced over the course of time to turn out to be completely incorporated into each component of the drug store. Administering of drugs, meeting, drug control, and the offer of these medications are instances of such cycles. In drug rehearses, local drug stores and clinic drug stores both play significant parts.

## 6. Proposed model for project

In this venture, the objective is to make a product parts library for multi-store medicine class data that is coordinated with the area’s drug stores. The pecking order, formal portrayal, and execution of every part will be pointed out overall, as well as the general and specific targets of the undertaking.

**Figure 1.**
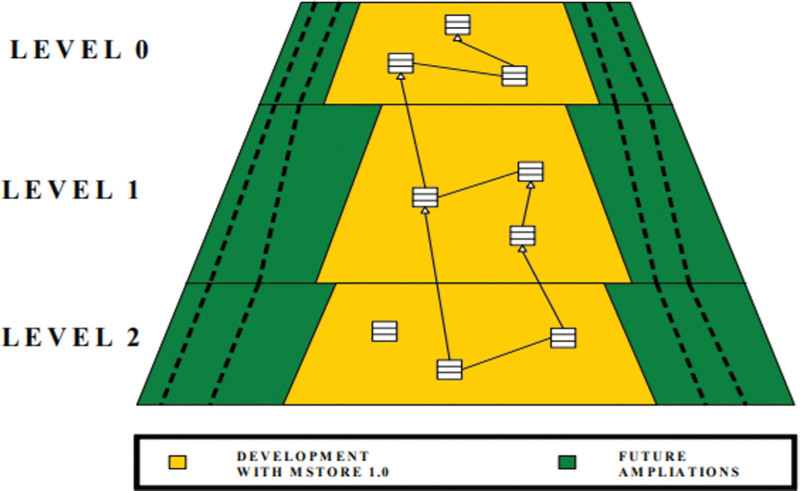
Multi category Drug project Scope.

Despite the fact that the plan endeavors to catch whatever number of angles would be prudent, the particular applications will just utilize a piece of it and completely confirm it. As displayed in Figure 1, the following undertakings will endeavor to extend the plan (looking for missing elements) and test the excess parts.

## 7. Analysis of project

As recently expressed, the framework ought to incorporate essentially the accompanying viewpoints at an exceptionally significant level:

Level 0: A connection point based structure that portrays the framework’s principal conduct and collaborations without requiring any execution.

Level 1: The most major execution of conduct that is considered widespread in all executions. Consider the legislative issues of the board, which depend on numerous quantitative techniques.

Level 2: Implementation of a customer’s answer, including store working strategies and different subtleties.

The reflection of the substances should be free of any execution detail, as the system endeavors to be just about as broad as could really be expected. The division of the structure plan and execution into 2 layers (connection points and conceptual classes) is considered basic separation. The third level is similarly significant since it will test the structure’s usefulness and give a few models for software engineers who are new to the system.

Since JHotDraw^[21]^ involves a realistic application plan and benefits, this is certifiably not a clever mapping.

**Figure 2.**
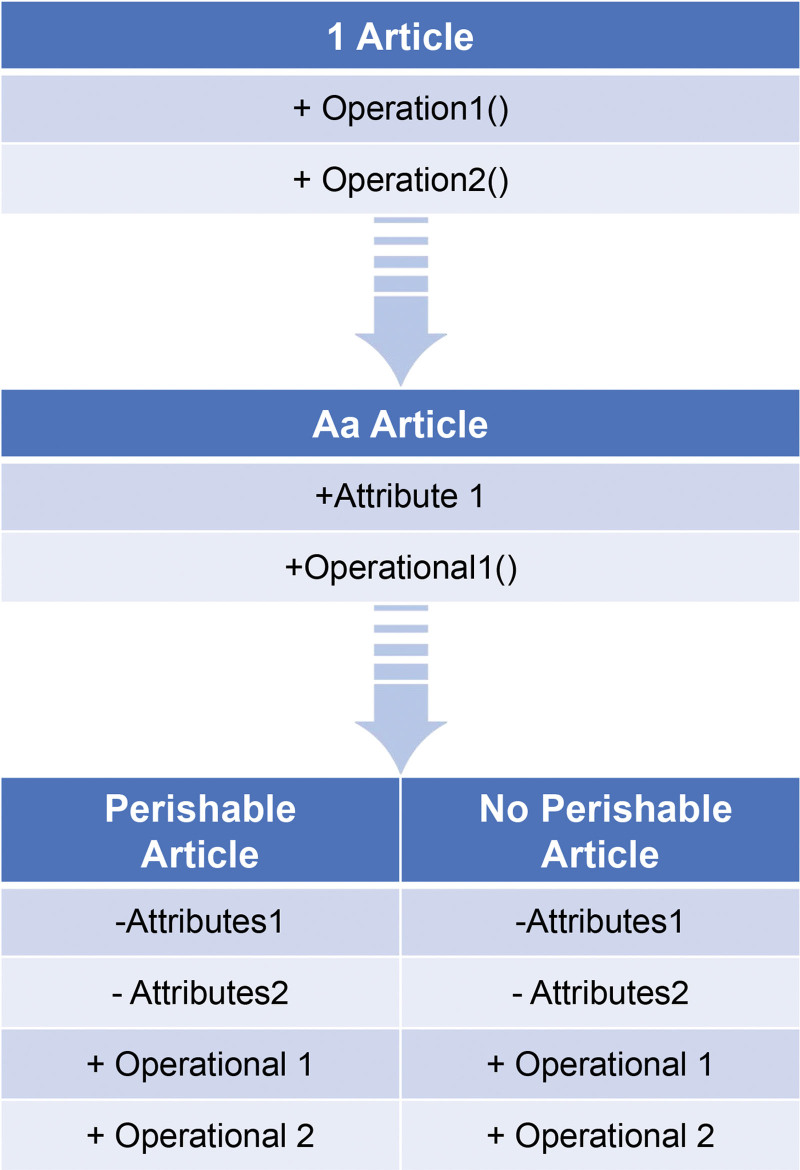
The three design levels in the abstraction “Article.”

For instance, going with Figure 2 tells the best way to fabricate every one of these pieces for this present reality reflection “Article” utilizing the procedure depicted. The reflection will be remembered for the point of interaction IArticle. Since the connections between interfaces at this level ought to never show signs of change, a great deal of work should be set into the plan of the points of interaction. The theoretical classes are executed at the second level with conduct that is normal for a gathering of articles. For instance, no matter what the sort of article is, the theoretical class Article can have every one of the elements that are normal to all articles. A third-level substantial class, like Perishable Article, is utilized by a substantial client in a particular application. Likewise, it is conceivable to construct new classes that are custom fitted to a specific circumstance. It can either reuse AArticle’s conduct supplier or change the activities to suit its requirements. Also, both making new activities and utilizing those that the connection point gives are available.

The following names will be used along the whole structure:

• Interfaces: < I > in addition to the name of the point of interaction.
• Unique classes: < A > in addition to the name of the theoretical class.
• Substantial classes: Simple name of the class.

Additionally, it will be endeavored to offer basic names to the deliberations in the main level, and more confounded names to the subclasses found in resulting levels whenever required.

## 8. The system’s requirements

The principal research in the subject of the executives of stores uncovered a profoundly different point.

Organizations that are keen on a program might have to change necessities relying upon an assortment of conditions, including the organization’s consideration and size, the items kept up with, the activities that happen in the store, the substantial format appropriation, etc.

It would consume a large chunk of the day to make a nonexclusive design that covers a wide range of stores. Likewise, it would turn out to be very troublesome when the techniques for making it conventional filled in size. Nonetheless, assuming the issue is delineated and only a couple of quantities of specific applications (under 10%) are barred, a nonexclusive structure might be made that covers 90% of the normal store worries while being extensively more straightforward to build and utilize.

Therefore, an expansive point of view of the framework uncovers that the executive structure of the store ought to incorporate the accompanying variables. Basically, this is an unpleasant aide; a more complete rundown of prerequisites has been aggregated, however it is excluded from the documentation:

## 9. Articles

Stock administration control, code the board, displaying of short-lived (took care of by sets and by sell-by date) and durable (dealt with independently) items, articles overseen by sets, ranges, families, inventories, evaluating and help control, dealt with units control

### 9.1. Entry and exit of merchandise

Everything connected with item passages in the store is made due, including conveyance zones, things differentiated by conveyance comments, and store design with signs of courses to use in the articles format.

Request readiness of the executives, packer the board, item get the executives (limiting developments to the racks, etc) are on the whole instances of item leave the executives at the store.

### 9.2. Order management

• Customary methods (least stock) and conventional stock administration rules are utilized to oversee stocks. Buying from providers, offering to customers, and overseeing different reports (spending plans, conveyance notes, tickets, solicitations, and so on)

Stock changes are allowed across zones and, furthermore, between stores, just as things are put away in the stores. It is basic to keep up with track of all data connected with these moves since it is important to know the status and area of every corporate thing (inventory...) consistently.

### 9.3. Management of resources

Representatives, gear, consumption, and using time effectively.

### 9.4. Store

• Deals with all parts of a store (execution should be multi-store)
• Format: It upholds many kinds of hardware, like Corridor Distribution.
• Conveyance: Some systems for finding free areas in the passage and going through the symmetric methodology on the way out are required. Contingent upon the zone, these systems might vary. When working with transient things, for instance, a FIFO system may provide some help.
• Actual Distribution: Ways of showing the store conveyance in a graphical organization. This will make it more straightforward for the client of things to come up with an application to control the total format.

### 9.5. Web services

All significant cycles ought to have a Web Service interface, bringing about a MVC plan that permits clients to get to the essential application functionalities through a few points of interaction, including Web Services.

### 9.6. Database management

Access is exemplified by an article social layer (data set admittance merchant).

The structure’s worked on the bundle chart might resemble this in its underlying form.

These considerations should be calculated into the structure’s engineering, which ought to be general to the point of considering future development. The peruser may accept that the undertaking may basically carry out a standard application, an executives application. Nonetheless, what will be fabricated is a conventional plan that the engineer can change the parts he doesn’t like (or that don’t meet his requirements), and adjust in the manner in which he needs. Undoubtedly, these structures are definitely more adaptable than a solitary program. The advantages and characteristics of the structures tend to be presented in the following areas.

## 10. Frameworks for object-oriented programming

A framework can be defined in a variety of structures can be characterized in an assortment of ways. Coming up next is a potential conventional definition:

“A structure is a gathering of classes that communicates a reusable plan of a program or a part of a program.”

This definition gives off an impression of being more fitting for the issue it is endeavoring to settle:

“A structure is a gathering of adaptable classes that depict an answer to a specific issue.”

Coming up next are the characteristics it looks for:

• It determines thes main deliberations just as their connection points.
• It builds up the items’ associations with each other.
• Utilizing redefinition, the structure is custom-made for explicit issues.
• Any default arrangement ought to be added.

Examples and Object-Oriented Frameworks are firmly associated since both permit reuse by catching viable programming improvement processes.

Systems center around reusing substantial thoughts, calculations, and executions in a specific programming language. Designs, then again, are worried about the reuse of unique plans. The example indicates how to tackle an issue, yet the structure determines the solution (or even an answer).

The most significant benefits of Object-Oriented Frameworks are listed as follows^[22]^:

Seclusion: Frameworks build up an unmistakable differentiation between plan and execution using points of interaction and conceptual classes. The points of interaction are steady, while the theoretical classes fuse execution changes that are more unpredictable. Furthermore, it’s simpler to appraise the expense of altering a few parts of the plan and execution, which eliminates the time and exertion important to comprehend and keep up with the program.

Reusability: Interfaces characterize conventional parts that might be used to fabricate new applications. Engineers can use the earlier endeavors to build their own plans since these endeavors have recently been delivered and checked. Reusing upgrades the proficiency of developers, just as the product’s quality, execution, and similarity.

Extensibility: A structure has express snare techniques that empower applications to upgrade the system’s steady connection points. Snare strategies separate from an application’s steady connection points and practices from the adjustments needed by launches in a specific climate in a calculated manner.

A Framework is a mix of the substantial and the theoretical since it is programming. Systems are more dynamic than most programming since they are reusable plans as opposed to coding or making documentation testing. Specialists in a specific space make structures, which are used by non-specialists. The ideal interest group for structure documentation consists of those who need to utilize it to resolve normal issues rather than those who just would like to make a product church. The crowd with demands reacts well to designs.

It is ordinarily recognized that portraying structures with designs is more advantageous. An essential objective of various examples is to show how to use a structure rather than how it functions, in spite of the fact that examples can likewise characterize a huge piece of the plan reasoning. Each example clarifies an issue that is average in the system’s concern space, trailed by guidelines on the best way to address it.

There are a great many similarities in the organization of each example. The most widely recognized strategy is to begin by depicting the circumstances. Following that, a full assessment of the different ways to deal with tackling the issue is introduced, utilizing models drawn from different segments of the structure. The response is summed up after completion of the example. Designs are issue centered rather than arrangement centered. Each example discloses how to address a particular part of a greater plan challenge. At times the arrangement is to make another subclass, different occasions it’s to define a current class’ item, and then again different occasions it’s to associate various articles together.

A structure’s documentation serves three goals, and examples can help with every one of them. It should incorporate the accompanying data:

• The system’s level-headed
• Step by step instructions to Put the Framework to Work
• The system’s complex plan.

Since designs are obviously appropriate for disclosing how to utilize a structure, an assortment of examples might satisfy every one of the three of these system documentation objectives.

## 11. JHotDraw

JHotDraw is a great illustration of how to portray a structure utilizing designs. It’s an exceptionally adjustable graphical UI system that makes making drawing applications a breeze. JHotDraw was roused by HotDraw, which was made by Kent Beck and Andre Winand, and introduced at OOPSLA97 by Thomas Eggenschwiler and Erich Gamma. It was made for a theological school to act as an illustration of how to involve designs in system improvement, yet the ideas might be utilized for proficient applications too.

**Figure 3.**
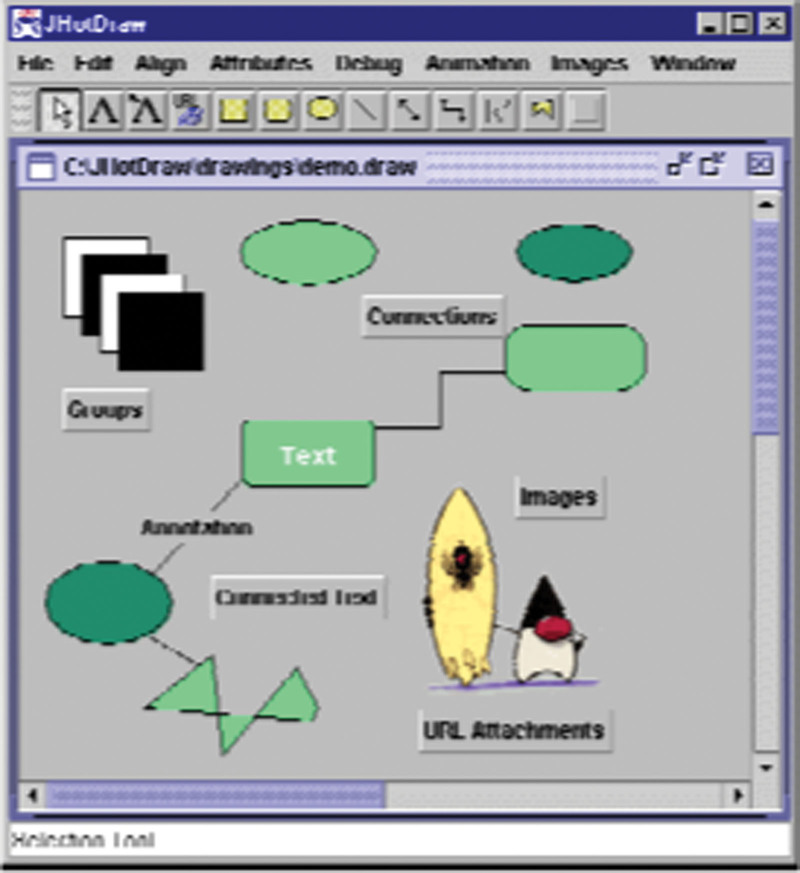
JavaDraw is a typical application of JHotDraw.

JHotDraw is a skeleton for a GUI-based editorial manager that remembers apparatuses for an instrument range, numerous viewpoints, client characterized graphical figures, and saving, stacking, and printing capacities (Fig. 3). The structure might be altered by blending and acquiring parts. Configuration Patterns with a programming stage like Java (and, as the peruser will find later,.NET) are a strong mix, while no language can remove the worth of a plan.

From a programming stance, the system is very captivating. It could very well be extended to the required usefulness or adjusted to current usefulness with some comprehension of JHotDraw’s design. The system is one of the earliest programming advancement activities to be marked as a structure and purposely worked for reuse. Additionally, it is one of the primary plan examples to be recorded, making it tremendously critical in the plan design local area. Composite, State, Template Method, Factory Method, and Strategy are the absolute most fundamental plan designs. Knowing basic central standards of such examples simplifies it to change the structure to match the requirements of a particular application.

## 12. Discussion

Previous studies have shown that no single management information system can effectively support the management of medicines in multiple hospitals.^[23]^ In Faradiba et al‘s research study, it was evident that the current pharmacy information system in Indonesia has limited functionality from the perspective of the pharmacist as a user.^[24]^ Therefore, searching for data about Drug Store Management necessities seems legitimate. On the one hand, it contributes to understanding the trouble of dealing with a store; and on the other hand, it delivers an answer that may make the board applications of stores more straightforward. Following some examination that exhibited the expansiveness of this subject, every one of the particulars was outlined and split between 2 understudies, each responsible for a gathering of bundles. The two understudies worked pair and teamed up to build the entire design.

Simultaneously, by finishing this activity, the fashioners will have a more profound comprehension of the issue and more confidence in completing the structure of MStore. This system will give the client the advantages referenced in the third segment of this study, just as a large number of different advantages in the field of store organization. Making an administration program might seem basic from the outset, yet as the venture fills in size, different arrangements that help the architect (plan designs, systems, technologies...) should be taken into consideration. From that point forward, it was a significant advantage to concentrate on O. O. Systems in more prominent profundity prior to setting out on one. JHotDraw was the design that was picked, and its configuration structure was set up by the examples.

With the advantages that plan designs offer us, we will actually want to accomplish a strong answer to every issue uncovered during the program investigation. With the advantages that plan designs manage the cost of us, since they have been tested commonly.

## 13. Conclusion

The design and implementation of an intelligent information management system for pharmacies is an integral part of the healthcare intelligence process. This study provides data on the best ways to create and implement a pharmacy management system. The board framework of a drug store with configuration annotations and descriptions has significantly shorter learning times for drug specialists, which is likewise beneficial for patients.

## Author contributions

**Conceptualization:** Qihong Pan.

**Investigation:** Yang Liu.

**Writing – original draft:** Shaofeng Wei.

**Writing – review & editing:** Shaofeng Wei.

## Correction

This article was originally published without funding information. Funding Information (grant number NYB22006 was added to the funding information for Nanchang Medical College Doctoral Research Initiation Fund Project) has now been added in the online version.
